# *Portulaca oleracea* L. (purslane) extract ameliorates intestinal inflammation in diet-induced obese mice by inhibiting the TLR4/NF-κB signaling pathway

**DOI:** 10.3389/fphar.2024.1474989

**Published:** 2025-01-07

**Authors:** Lingchao Miao, Meng Sam Cheong, Haolin Zhang, Haroon Khan, Hongxun Tao, Yuxiao Wang, Wai San Cheang

**Affiliations:** ^1^ State Key Laboratory of Quality Research in Chinese Medicine, Institute of Chinese Medical Sciences, University of Macau, Macau SAR, China; ^2^ Department of Pharmacy, Abdul Wali Khan University Mardan, Mardan, Pakistan; ^3^ Department of Pharmacy, Korea University, Sejong, South Korea; ^4^ School of Food and Biological Engineering, Jiangsu University, Zhenjiang, China; ^5^ College of Food Science and Engineering, Shandong Agricultural University, Tai’an, Shandong, China

**Keywords:** purslane, obesity, intestine, inflammation, NF-κB pathway

## Abstract

**Background:**

*Portulaca oleracea* L. (purslane) is a dietary plant and a botanical drug with antioxidant, antidiabetic, and anti-inflammatory activities. However, the effects of purslane against intestinal-inflammation-associated obesity are yet to be studied. In the present study, we hypothesized that purslane extract could reduce intestinal inflammation associated with metabolic disorder.

**Results:**

Male C57BL/6J mice were fed a high-fat diet (HFD, 60% kcal% of fat) for a total duration of 14 weeks to establish an obesity model; further, the treatment group was orally administered purslane extract (200 mg/kg/day) during the last 4 weeks. Then, intestinal tissues were detached from the mice for detecting protein expressions through Western blot and immunohistochemical analyses. Pro-inflammatory cytokines were determined using ELISA kits, whereas the components of purslane extract were detected by ultra performance liquid chromatography/electrospray ionization quadrupole time-of-flight mass spectrometry. Chronic oral administration of purslane extract ameliorated colon shortening syndrome and reduced bowel inflammation in HFD-induced obese mice through suppression of the toll-like receptor 4 (TLR4)/nuclear factor kappa B (NF-κB) signaling pathway to downregulate TLR4, myeloid differentiation factor 88 (MyD88), Ser32 phosphorylation of NF-κB inhibitor alpha (IκBα), and Ser536 phosphorylation of NF-κB p65 expression levels, thereby inhibiting the pro-inflammatory cytokines, tumor necrosis factor (TNF)-α and interleukin (IL)-6 levels.

**Conclusion:**

The present study supports the anti-inflammatory potential of purslane extract for modulating bowel inflammation under obesity through inhibition of the TLR4/NF-κB signaling pathway.

## Background

Metabolic disorders such as obesity, type 2 diabetes mellitus, and cardiovascular diseases are interconnected and often associated with long-term high-fat dietary habits (Kratz et al., 2013). Furthermore, diabetes and obesity are characterized by chronic inflammation throughout the body, including the intestinal system (Calder et al., 2011; Bleau et al., 2015). Chronic systemic inflammation can lead to the recruitment of immune cells like T lymphocytes and macrophages, which then results in the production of pro-inflammatory cytokines such as tumor necrosis factor (TNF)-α and interleukin (IL)-6. The toll-like receptor 4 (TLR4) signaling pathway is a major trigger of inflammatory responses stimulated by obesity (Rogero and Calder, 2018). Upon receiving stimulus signals, the TLR4 activates the myeloid differentiation factor 88 (MyD88) and interacts with intracellular adapters to promote the activation of transcription factors, such as nuclear factor kappa B (NF-κB), which in turn induces gene transcription (Gorina et al., 2011).

Growing evidence has implicated the intestinal immune system as an important contributor to metabolic diseases, including obesity and diabetes (Khan et al., 2021). Gut tissues comprise an extensive immune system that can be affected by exposure to microbial antigens and ingested antigens from daily dietary sources (Garidou et al., 2015; Lu et al., 2024). In-depth investigations have shown that inflammatory and immune cell changes in the bowels are involved in the pathology of obesity and insulin resistance (Luck et al., 2015; Monteiro-Sepulveda et al., 2015). Therefore, it is of great significance to explore efficient medicines that can regulate long-term and excessive immune responses to ultimately reduce inflammation of the intestinal tissues.

*Portulaca oleracea* L. (purslane) is a natural plant used widely around the world as both food and traditional medicine (Uddin et al., 2014). The major constituents identified in purslane are flavonoids, fatty acids, polysaccharides, alkaloids, proteins, sterols, vitamins, and minerals (Nemzer et al., 2020). These components have been reported to exhibit multiple bioactivities, including antiobesity, antidiabetic, anti-inflammatory, antioxidant, vasoprotective, and antitumor effects (Petropoulos et al., 2016; Miao et al., 2019, 2023). However, specific information on the bioactivity of purslane for relieving intestinal inflammation in obesity as well as the underlying mechanisms remains deficient and unclear.

In the present study, we aimed to investigate the anti-inflammatory effects of purslane extract on obesity-associated chronic intestinal inflammation *in vivo* using high-fat diet-induced obese (DIO) mice to elucidate the mechanisms involved in the observed anti-inflammatory effects.

## Materials and methods

### Preparation of purslane extract

*Portulaca oleracea* L. plants were purchased from Huangshan, Anhui Province, China. Ten grams of the dried plant materials was smashed equably and soaked in 100 mL of 50% ethanol at 25°C in a sealed beaker for 24 h in the dark. Then, the soaked solution was collected, and the powder was extracted twice with a fresh batch of 100 mL of 50% ethanol for 2 h by ultrasonic treatment at 100 W. Thereafter, the two extracts were mixed, filtered by suction, and concentrated into a thick extract without excess water and ethanol using a rotary evaporator at a low speed and 70°C. High-performance liquid chromatography (HPLC) grade ethanol was purchased from RCI Labscan Limited, Pathumwan, Bangkok, Thailand), and Milli-Q water was prepared using a Milli-Q purification system (Millipore, MA, United States). The concentrated extract was stored overnight at -80°C, followed by freeze-drying with a VirTis freeze dryer (VirTis Company, New York, United States) for 2 d to obtain the freeze-dried powder extract of purslane.

### Identification of purslane extract

The purslane extract was identified using ultra performance liquid chromatography/electrospray ionization quadrupole time-of-flight mass spectrometry (UPLC-ESI-QTOF-MS/MS; Waters, MA, United States). The liquid chromatographic separations were performed on a Waters Acquity UPLC H-Class system (Waters, MA, United States) equipped with a Waters Acquity UPLC BEH C18 column (2.1 × 50 mm, 1.7 µm), with the column temperature set to 30°C. The mobile phase consisted of 0.1% formic acid in water (phase A) and acetonitrile (phase B). HPLC grade acetonitrile was purchased from Merck (Darmstadt, Germany). The flow rate was set to 0.3 mL/min, and the injection volume was 1 µL. The samples were eluted with a linear gradient: 5%B for 0–1 min; 5%–95%B for 1–28 min; 95%–5%B for 28–36 min; 5%B for 36–40 min.

The MS analysis was performed on a Waters Xevo G2-XS QTOF system combined with an electrospray ionization (ESI) source (Waters, MA, United States). The mean-squared error centroid mode was used for sensitivity and negative ESI mode was used to collect fragment ions within the m/z range of 10–1,500 with the scan time set to 1 s. The tuning parameters were set to a capillary voltage of 2 kV, sampling cone voltage of 40 V, source offset of 80 V, source temperature of 120°C, desolvation temperature of 450°C, cone gas flow rate of 50 L/h, and desolvation gas flow rate of 700 L/h. The data were processed using MassLynx V 4.1 software (Waters).

## Animal experimental protocols

Male C57BL/6J mice (18–24 g) were provided by the Animal Centre of the University of Macau (Faculty of Health Science). This study received ethical approval from the University of Macau Animal Research Ethics Committee (UMARE-024-2021), in addition to following all the guidelines outlined in the Guide for the Care and Use of Laboratory Animals. All mice were cultivated under a controlled temperature of 22°C–24°C and 50% relative humidity with 12/12-h light/dark cycles. At 8 weeks of age, the mice were randomly divided into three groups as control, DIO, and DIO treated with purslane (four mice per group). The control group was fed a standard chow diet, while the other mice were given a high-fat diet (HFD; 60% kcal% of fat; Shuyishuer Bio, Changzhou, China) for a total of 14 weeks along with free access to water. The DIO treatment group was orally administered purslane extract (200 mg/kg/day dissolved in 0.3% carboxymethyl cellulose sodium), whereas the other mice from the control and DIO groups were administered a vehicle solution during the last 4 weeks. Thereafter, all groups of mice were euthanized and intestinal tissues were collected.

### Pro-inflammatory cytokine determination

The intestinal tissues were obtained from all groups of mice, divided into large and small intestine parts, frozen with liquid nitrogen, and stored at −80°C. Approximately 1.5-cm-length tissues samples from the same part of the colon were homogenized using saline and precooled instruments. The supernatant was collected after the homogenate was centrifuged for 10 min at 2,500 rpm/min and 4°C. The IL-6 and TNF-α concentrations were determined in all mice intestinal tissues using ELISA kits following the manufacturer’s instructions (NeoBioscience, Shenzhen, China).

### Western blotting analysis

Protein concentrations in the colon tissue lysates were measured via the bicinchoninic acid (BCA) assay (ThermoFisher Scientific, MA United States). Equal amounts of samples were resolved using sodium dodecyl sulfate polyacrylamide gel electrophoresis (SDS-PAGE) and transferred to a polyvinylidene fluoride (PVDF) membrane (Millipore Corp., MA, United States) on ice by the wet transfer mode (BIO-RAD, United States). After blocking with 5% defatted milk and tris-buffered saline (TBS) containing 0.1% Tween-20, the membrane was probed with appropriate primary antibodies diluted to 1:1,000 against TLR4, MyD88, phosphorylated NF-κB inhibitor alpha (IκB⍺) at Ser32, total IκB⍺, phosphorylated p65 at Ser536, total p65, and β-actin (Cell Signaling Technology, United States) overnight at 4°C. The protein bands were then scanned using the ChemiDocTM MP imaging system (BIO-RAD, United States) after incubation with corresponding secondary antibodies (Cell Signaling Technology, United States) using the Supersignal TM West Femto maximum sensitivity substrate (ThermoFisher Scientific, PA, United States). The band intensities were quantified using Image Lab software (Version 3.0).

### Histological analysis

Small intestine samples from all mice from roughly the same locations were collected and fixed in 4% paraformaldehyde solution. Then, the samples were embedded in paraffin and sliced into 5-μm-thick sections before being stained with hematoxylin and eosin (H&E; Beyotime Biotechnology, Shanghai, China). Additionally, immunohistochemistry (IHC) analyses of IL-6, TNF-α, TLR4, and phosphorated p65 (Ser536) were conducted as described by Liu et al. (2019). The paraffin sections were dewaxed, and the antigens were retrieved with EDTA (pH 9.0) and gradient organic solvents, followed by 2-h of blocking with 5% bovine serum albumin (BSA) and TBS with Tween-20 as well as blocking with 3% H_2_O_2_ (aq) for 25 min at room temperature. Next, the sections were incubated with the primary antibodies of IL-6, TNF-α, TLR4, and phosphorated p65 at Ser536 (1:100, Cell Signaling Technology, United States) overnight at 4°C, followed by incubation with the corresponding secondary antibodies. The staining was determined using an I-View DAB detection system (Servicebio, Beijing, China), and the sections were stained with hematoxylin (Beyotime Biotechnology, Shanghai, China) to visualize the nuclei before being dehydrated. All the stained specimens were observed and recorded using an Olympus IX73 microscope. The H&E sections were photographed at ×40 and ×200 magnifications, and the IHC sections were photographed at ×400.

### Statistical analysis

The data were recorded as mean ± standard error of the mean (SEM) for all experiments. For the statistical analysis, GraphPad Prism (Version 9.1.1, United States) was used to obtain multiple comparisons between the experimental groups by one-way ANOVA and unpaired Student’s *t*-test. A *p*-value <0.05 was deemed to be significant.

## Results

### Identification of purslane extract

**FIGURE 1:**
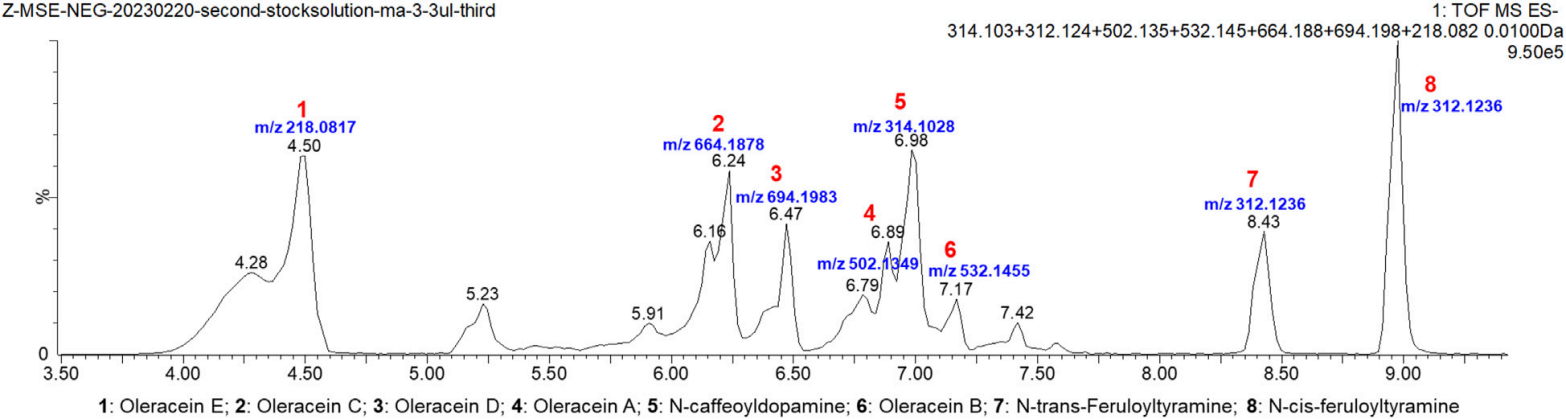
Extracted ion chromatogram (EIC) of the eight amide components identified in purslane extract, namely, oleracein A, oleracein B, oleracein C, oleracein D, oleracein E, N-caffeoyldopamine, N-trans-feruloyltyramine, and N-cis-feruloyltyramine.

Eight amide components were identified from the prepared purslane extract, which are shown in the extracted ion chromatogram (EIC) in Figure 1. The information on the identification of the eight components in the purslane extract by ESI-QTOF-MS/MS in the negative ESI mode is shown in Table 1, including the retention time, observed mass, theoretical mass, predicted chemical formula, and observed MS/MS fragment ions. The identification results and bases are also listed in Table 1. Here, N-trans-feruloyltyramine matched with the MS/MS result in the PubChem database; oleracein A, B, C, D, and E along with N-caffeoyldopamine were identified by referring to the MS/MS information in literature (Jiao et al., 2014); lastly, N-cis-feruloyltyramine was identified only using the exact mass in this study.

**TABLE 1:** Identification information regarding the eight components found in purslane extract by ESI-QTOF-MS/MS. The retention time, observed mass, theoretical mass, predicted chemical formula, observed MS/MS fragment ions, identification results, and basis of each of the eight components are listed.

Compound No.	Retention time (min)	Observed mass, m/z	Theoretical mass, m/z	Predicted chemical formula	Error (ppm)	Observed MS/MS fragment ions	Method of identification	Chemical compound name/CAS No.
1	4.50	218.0819	218.0817	C_12_H_13_NO_3_	−0.9	218.0821, 162.0558, 135.0446, 188.0756,	Jiao et al. (2014)	Oleracein E (+/−)-Trolline 1,021,950–79-7
2	6.24	664.1887	664.1878	C_30_H_35_NO_16_	1.4	518.1516, 340.0840, 664.1797, 194.0501, 296.0955		Oleracein C
3	6.47	694.1979	694.1983	C_31_H_37_NO_17_	−0.6	518.1489, 694.1913, 370.0941, 335.1257, 194.0501		Oleracein D
4	6.89	502.1376	502.1349	C_24_H_25_NO_11_	5.4	145.0292, 342.1350, 194.0468, 298.1075, 252.1080		Oleracein A
5	6.98	314.1039	314.1028	C_17_H_17_NO_5_	3.5	135.0449, 152.0715, 161.0244, 178.0503, 122.0364		N-caffeoyldopamine 103,188–49-4
6	7.17	532.1467	532.1455	C_25_H_27_NO_12_	2.3	175.0446, 370.0944, 194.0502, 326.1043		Oleracein B
7	8.43	312.1241	312.1236	C_18_H_19_NO_4_	1.6	148.0526, 178.0504, 190.0505, 135.0443	PubChem database	N-trans-feruloyltyramine 66,648–43-9
8	8.98	312.1242	312.1236	C_18_H_19_NO_4_	1.9	148.0528, 178.0506, 190.0508, 300.0277, 135.0448	Only exact mass	N-cis-feruloyltyramine 80,510–09-4

### Purslane treatment alleviated colon shortening caused by obesity in DIO mice

**FIGURE 2:**
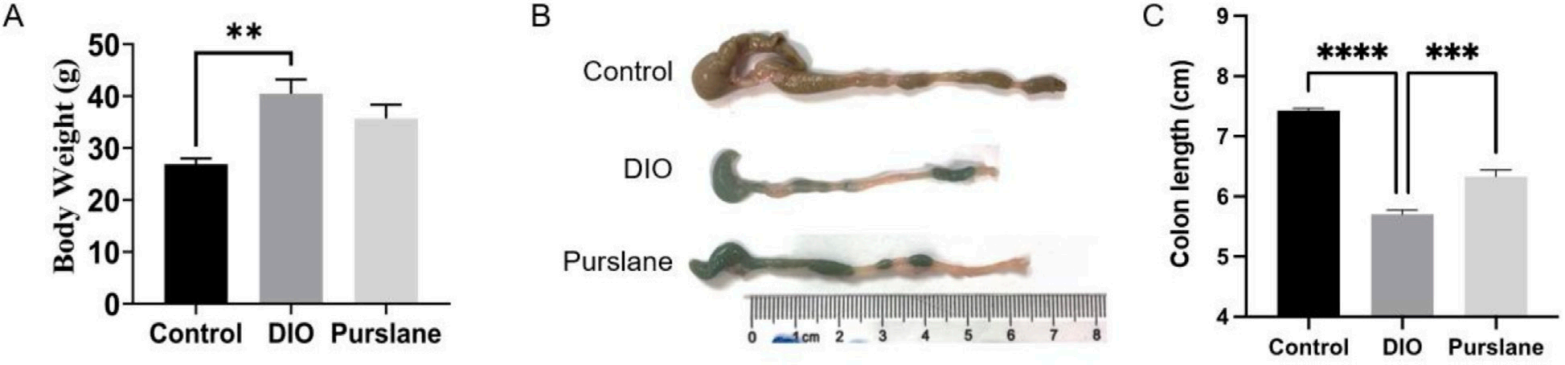
Purslane extract treatment alleviates colon shortening in DIO mice: **(A)** bodyweights of mice fed high-fat or standard chow diets for a total of 14 weeks and administered either the vehicle (0.3% CMC-Na solution) or purslane extract (200 mg/kg/day) dissolved in vehicle solution by oral gavage during the last 4 weeks; **(B, C)** representative images and summarized graph showing that chronic treatment with purslane extract ameliorates colon shortening in DIO mice. The data are reported as mean ± SEM of four mice. ∗*p* < 0.05, ∗∗∗<0.001, ∗∗∗∗<0.0001 for statistical significance.

The DIO mice were administered purslane extracts at 200 mg/kg/day by oral gavage for a total duration of 4 weeks to investigate its anti-inflammatory effects on intestinal tissues *in vivo*. Before commencing the experiments, the mice had similar bodyweights and were randomly assigned into different groups. As shown in Figure 2A, after the 14-week HFD induction, the final bodyweights of the DIO mice were much higher than those of the control mice that received a standard chow diet, indicating successful establishment of obese mouse model. Interestingly, the 4-week oral administration of purslane extract seemed to produce a slight but non-significant weight-reducing effect on the DIO mice. Figure 2B shows that the colon length of a representative DIO mouse was obviously shorter (5.5 cm) than that of the control mouse (7.2 cm), which was significantly improved by chronic purslane treatment (6.4 cm). The summarized graph of the colon lengths of all groups of mice is shown in Figure 2C, indicating that purslane extract alleviates colon shortening in DIO mice.

### Purslane treatment suppressed pro-inflammatory cytokine levels in the intestinal tissues of DIO mice

**FIGURE 3:**
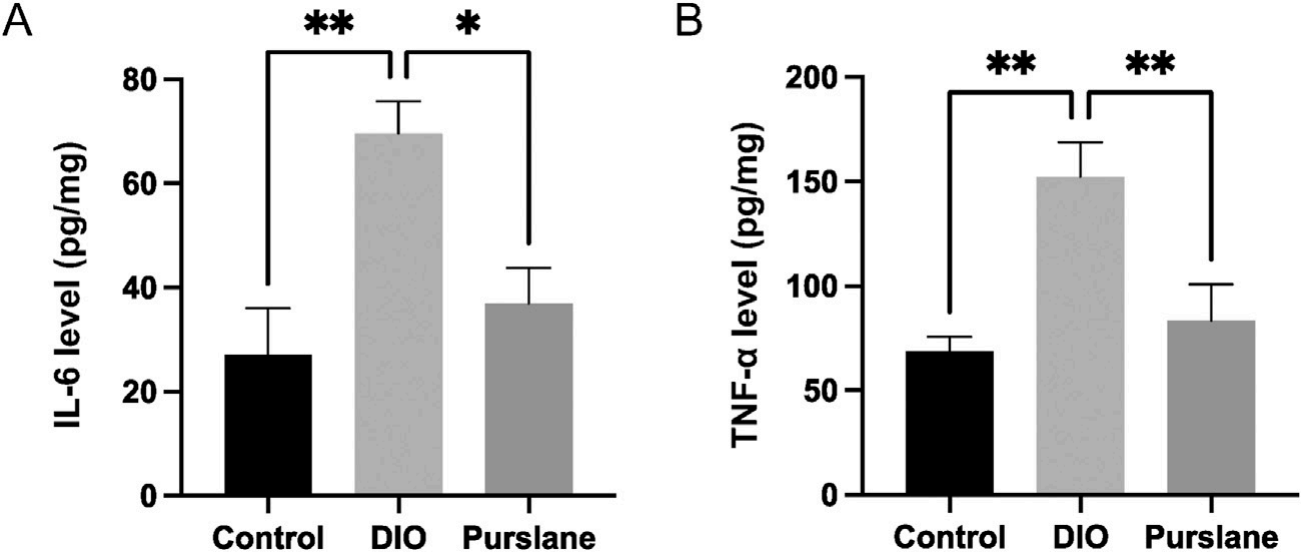
Effects of purslane extract on pro-inflammatory cytokine levels in colon tissues from DIO mice: **(A)** IL-6 and **(B)** TNF-α expression levels in the intestinal tissues of mice after chronic treatment. The data are reported as mean ± SEM of four mice. ∗*p* < 0.05, ∗∗*p* < 0.01 for statistical significance.

To investigate the anti-inflammatory capabilities of purslane extract, the expression levels of pro-inflammatory cytokines IL-6 and TNF-α in the colon tissues were determined using ELISA kits. The IL-6 and TNF-α levels were upregulated sharply in the DIO mice but reversed significantly after purslane treatment to levels comparable to those of the control group (Figures 3A, B). These results illustrate that a 4-week oral administration of purslane extract (200 mg/kg/day) could suppress intestinal inflammation in DIO mice.

### Purslane extract inhibited colon inflammation in DIO mice by suppressing the TLR4/MyD88/NF-κB signaling pathway

**FIGURE 4:**
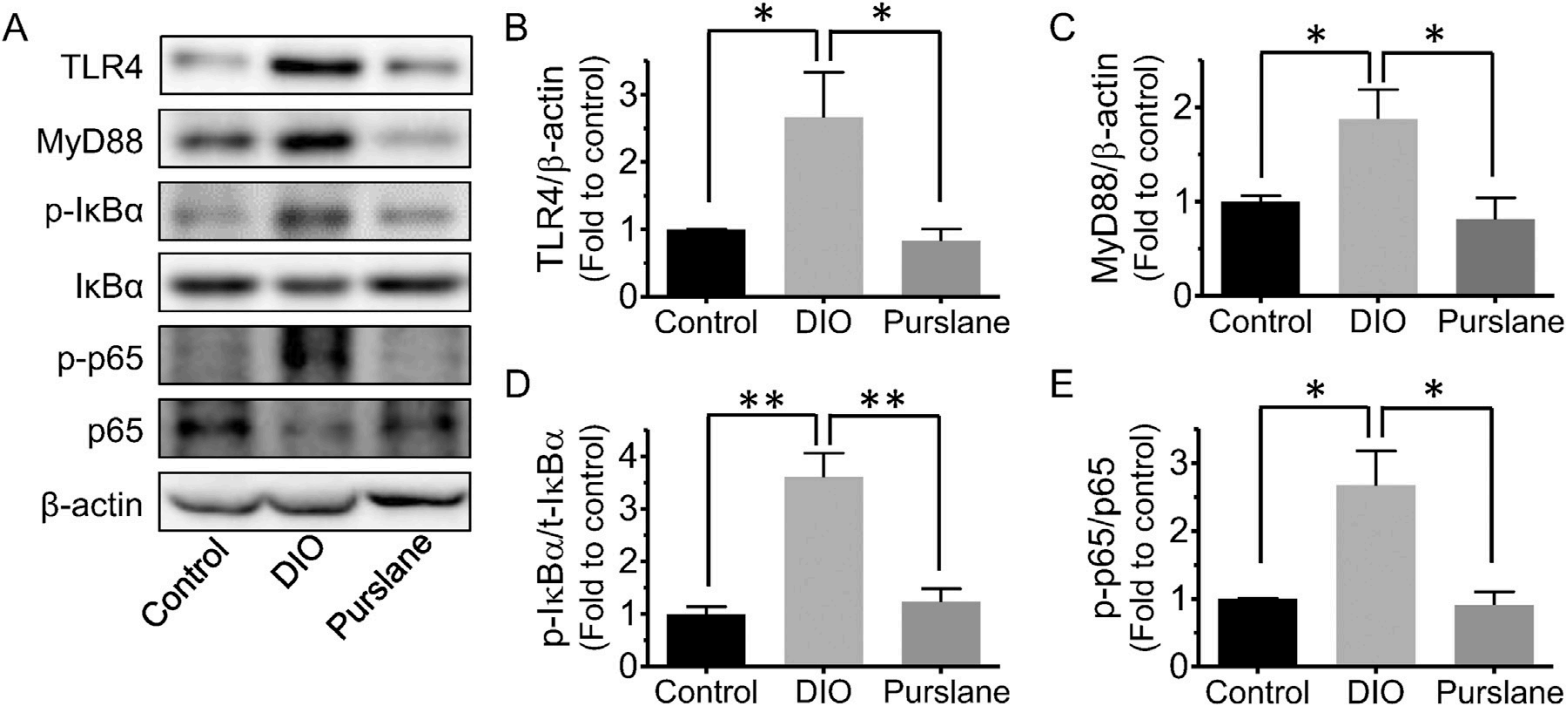
*In vivo* treatment with purslane extract suppresses the TLR4/MyD88/NF-κB pathway in the colons of DIO mice: **(A)** representative bands of Western blotting. Summarized graphs showing the expression levels of **(B)** TLR4, **(C)** MyD88 compared to β-actin, **(D)** phosphorylation at Ser32 and total IκBα, and **(E)** phosphorylation at Ser536 and total p65. The data are reported as mean ± SEM of four mice. ∗*p* < 0.05, ∗∗*p* < 0.01 for statistical significance.

Colon tissues of all groups of C57BL/6J mice were obtained to further study the underlying action mechanisms of purslane by Western blotting. It was discovered that compared to the control group, the TLR4, MyD88, Ser32 phosphorylation of NF-κB inhibitor alpha (IκBα), and Ser536 phosphorylation of NF-κB p65 expression levels (compared to β-actin or related total proteins) were all remarkably upregulated in the colons of DIO mice (Figures 4A–D). Purslane extract treatment significantly reduced the protein expressions of TLR4 and MyD88 as well as the phosphorylation of IκBα and NF-κB p65 in the colon sections, suggesting inactivation of the TLR4/MyD88/NF-κB signaling pathway and contributing to inflammation relief.

### Purslane extract improved bowel histopathological injury in DIO mice

**FIGURE 5:**
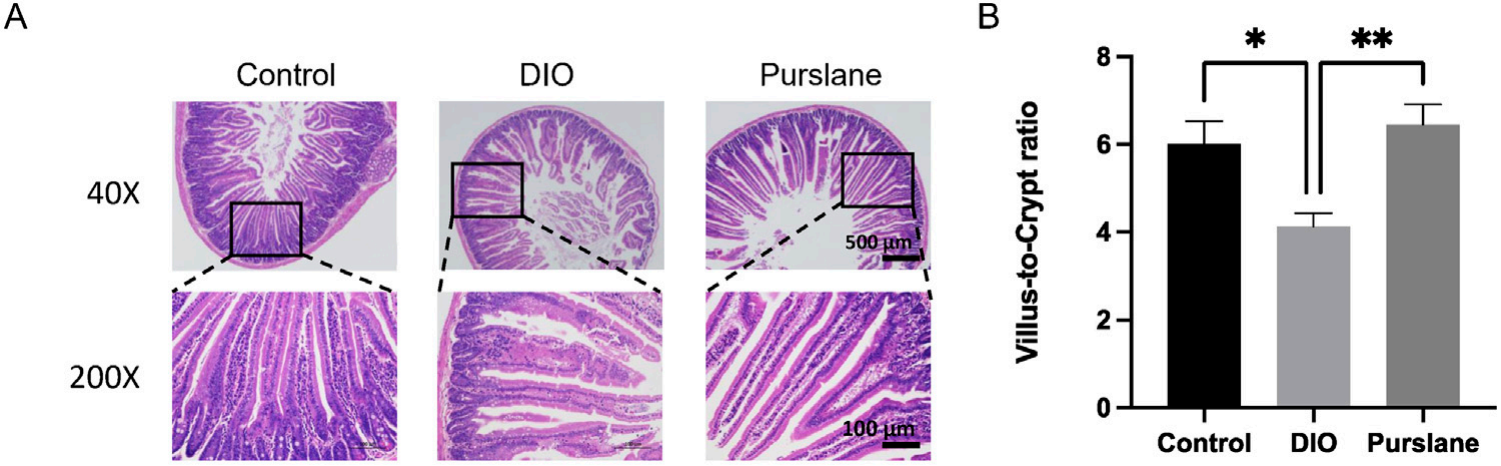
Chronic treatment with purslane extract improves bowel histopathological injury in DIO mice: **(A)** representative H&E staining images of small intestine sections (magnification at ×40 and ×200; scale bar = 500 μm and 100 µm); **(B)** summarized graph representing the ratio of villus length to crypt depth in all groups of mouse small intestine tissues. The data are reported as mean ± SEM (n = 4). ∗*P*< 0.05, ∗∗*p* < 0.01 for statistical significance.

H&E staining was used to observe the histopathological changes in the mouse small intestine tissue, and the corresponding experimental results are shown in Figure 5. The intestinal mucosal structures of the mice in the control group had normal morphologies, with shallow crypts, neatly arranged intestinal villi, and obvious boundaries. In the DIO group, the intestinal villi were disordered with missing top structures, deep crypts, and indistinct boundaries, and a part of the intestinal cortex was separated from the lamina propria. Nevertheless, 4-week oral administration of purslane extract was observed to significantly improve the intestinal injury status and prevent reduction of the ratio of intestinal villus length to crypt depth (*p* <0.01) in the DIO mice (Figures 5A,B); this ratio of villus length to crypt depth is an important index for evaluating the structural integrity of the small intestinal mucosa. To conclude, chronic treatment with purslane extract was found to improve bowel histopathological injury in DIO mice effectively.

### Purslane extract inhibited inflammation in the small intestines of DIO mice by suppressing the TLR4/NF-κB pathway

**FIGURE 6:**
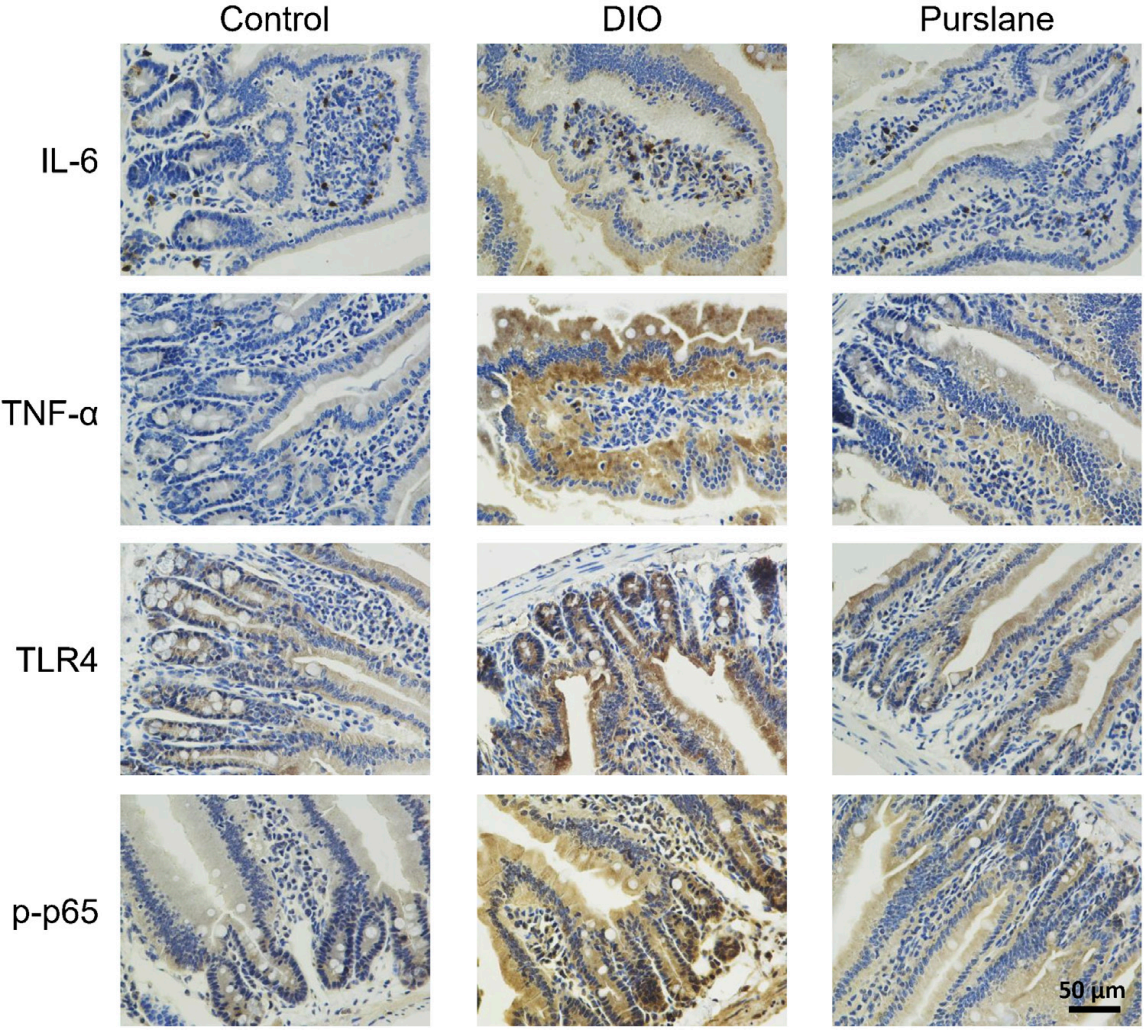
Oral administration of purslane extract ameliorates inflammation by inhibiting IL-6, TNF-α, TLR4, and phosphorylated NF-κB p65 expression levels in the small intestines of DIO mice. Representative immunohistochemical (anti IL-6, TNF-α, TLR4, and Ser536 phosphorylated NF-κB p65) images of small intestines from the control, DIO, and DIO treated with purslane extract groups (magnification at ×400; scale bar = 50 µm).

In line with the results of the inflammatory marker measurements in the mouse colons, IHC analysis of the small intestine tissues illustrated elevated expression levels (indicated by brown color) of IL-6, TNF-α, TLR4, and phosphorylated NF-κB p65 (Ser536) in the DIO mice and that such elevation was lowered after oral treatment with purslane extract (Figure 6). This phenomenon demonstrates that purslane extract ameliorates intestinal inflammation by suppressing the TLR4/NF-κB pathway in the small intestine tissues of DIO mice.

## Discussion

In the present research, the anti-inflammatory and intestinal protective functions of purslane were verified in HFD-induced obese and diabetic mice, which have not been verified until now. The novel findings of this study include the following: (i) long-term treatment with purslane ameliorates colon shortening syndrome and the structural integrity of the small intestinal mucosa; (ii) suppression of the TLR4/NF-κB signaling pathway; (iii) reduced levels of pro-inflammatory cytokines IL-6 and TNF-α in both the small intestines and colons of DIO mice.

In our previous study, purslane extract was used to treat lipopolysaccharide-stimulated RAW 264.7 macrophages and was found to exert anti-inflammatory effects through suppression of the mitogen-activated protein kinase (MAPK) and NF-κB signaling pathways (Miao et al., 2019). It was also verified that the aqueous extract of purslane possesses protective effects against hyperglycemia, inflammation, and oxidative stress in the sera of streptozotocin (STZ)-induced diabetic rats (Samarghandian et al., 2017). The purslane stem has also been reported to attenuate serum total lipid, total cholesterol, triglyceride, low-density lipoprotein (LDL) cholesterol, and very-low-density lipoprotein (VLDL) cholesterol as well as increase high-density lipoprotein (HDL) cholesterol levels in HFD-induced hyperlipidemic Wister rats (El-Newary, 2016). Importantly, clinical trials have provided consistent evidence illustrating that freeze-dried purslane supplements could help hypercholesterolemic adults lower their blood lipids (plasma total and LDL cholesterol levels) as well as significantly increase HDL cholesterol levels (Samuel et al., 2011; Sabzghabaee et al., 2014). Purslane extracts have also been shown to inhibit the levels of pro-inflammatory cytokines (TNF-α, 1L-1β, and IL-6) in mice suffering from dextran sodium sulfate (DSS)-induced colitis (Kim et al., 2018). Nonetheless, the present study is a pioneering effort at illustrating that bowel inflammation in DIO mice could be ameliorated using purslane extracts through suppression of the TLR4/NF-κB signaling pathway. The reduced production of IL-6 and TNF-α contribute to the anti-inflammatory activities of purslane extract. Moreover, eight amides were identified in the purslane extract, especially relatively substantial amounts of oleracein A, B, C, D, and E. The detected classes of chemical and oleracein components should be investigated further in a future study for their roles in the anti-inflammatory effects of purslane.

The intestinal tract is responsible for not only food digestion and nutrient absorption but also barrier activity to prevent intestinal pathogenic bacteria and endotoxins from invading the body (Farré et al., 2020). A complete intestinal mucosal barrier is critically important as it separates the intestinal cavity from the internal environment of the body to prevent harmful substances or pathogens in the intestinal cavity from moving into the external tissues and organs (Tlaskalová-Hogenová et al., 2011). Studies have found that damage to the intestinal mucosal barrier is associated with obesity, inflammatory bowel disease, diabetes, and other adverse health conditions (Michielan and D’Incà, 2015; Cheru et al., 2019). Our current study provides evidence on the nutritional interventions of purslane extract to maintain the integrity of the intestinal mucosal barrier and ameliorate the occurrence of bowel inflammation.

The TLR4/MyD88/NF-κB signaling pathway is a classical inflammatory pathway that regulates the expression levels of downstream pro-inflammatory cytokines, such as TNF-α and IL-6. The TLR4/MyD88 signaling pathway is stimulated in the intestinal tissue of DIO mice, which then initiates a downstream signaling cascade to cause phosphorylation and degradation of IκBα, resulting in the translocation of two subunits of NF-κB (p65 and p50) that enter the nucleus to induce transcription of inflammatory factor genes by binding to specific regions of the target genes (Li et al., 2020; Li and Verma, 2002). The expressions and releases of TNF-α, IL-6, and other pro-inflammatory cytokines and inflammatory mediators are promoted, which mediate the development of intestinal injury (Umesalma and Sudhandiran, 2010). The present study investigated HFD-induced intestinal inflammation and injury by dissecting and analyzing the length of the colon from different groups of mice, histochemical analysis of the intestines like HE staining, as well as IHC, Western blotting, and ELISA. Inflammation was obviously noted in the intestinal tissues of DIO mice, which manifested as significantly shortened colon length, damaged villi structure in the small intestine, and upregulated expressions of the inflammatory markers. Importantly, 4-week oral gavage of purslane extract was found to effectively alleviate the aforementioned intestinal inflammation and injury in DIO mice. In agreement with previous studies, the experimental findings of this study demonstrate that obesity significantly increases the expression levels of TLR4 and MyD88 proteins as well as the phosphorylation levels of IκBα and NF-κB p65, along with pro-inflammatory cytokine levels in the intestinal tissues of DIO mice. These inflammatory responses could be effectively inhibited by administration of purslane extract.

## Conclusion

In the present study, an obesity model of intestinal inflammation was established to systematically investigate the protective effects and underlying action mechanisms of purslane extract *in vivo*. Four-week oral administration of purslane extract was shown to confer protection against intestinal inflammation *via* downregulation of the TLR4/MyD88/NF-κB signaling pathway through lowered pro-inflammatory cytokine (IL-6 and TNF-α) levels in DIO mice. The findings of this study are a pilot effort at providing a scientific basis to support the potential use of purslane for preventing intestinal inflammation associated with diabetes and obesity. These results contribute to the growing body of evidence that highlights the health benefits of purslane and its potential as a health supplement or therapeutic agent.

## Data Availability

The original contributions presented in this study are included in the article/supplementary material, and any further inquiries may be directed to the corresponding author.
